# Efficacy of (−)-epigallocatechin gallate delivered by a new-type scaler tip during scaling and root planing on chronic periodontitis: a split-mouth, randomized clinical trial

**DOI:** 10.1186/s12903-021-01418-1

**Published:** 2021-02-18

**Authors:** Yanfeng Wang, Jiajun Zeng, Qiao Yuan, Qingxian Luan

**Affiliations:** Department of Periodontology, Peking University School and Hospital of Stomatology, National Engineering Laboratory for Digital and Material Technology of Stomatology, Beijing Key Laboratory of Digital Stomatology, No. 22, Zhongguancun South Avenue, Haidian District, Beijing, China

**Keywords:** (−)-Epigallocatechin gallate, New-type scaler tip, Subgingival scaling, Chronic periodontitis, Red complex pathogens

## Abstract

**Background:**

(−)-Epigallocatechin Gallate (EGCG) as green tea catechins possessed antibacterial and anti-inflammatory effects on periodontal disease. This study was designed to evaluate the clinical and microbiological efficacy of scaling and root planing (SRP) using EGCG aqueous solution as coolants through a new-type ultrasonic scaler tip on chronic periodontitis.

**Methods:**

This split-mouth, randomized clinical trial included 20 patients (2 drop-outs) with chronic periodontitis and the maxillary contra-lateral sides were allocated into test and control groups randomly. Through the new-type scaler tip, 762 sites with probing depth (PD) ≥ 4 mm were treated by SRP using EGCG solution or distilled water as coolants respectively. Clinical parameters and red complex pathogens in subgingival microbiome were evaluated at baseline, 3 and 6 months after treatments.

**Results:**

During 6 months, the SRP plus EGCG medication contributed to additional PD reduction as 0.33 mm and gain of clinical attachment level as 0.3 mm compared with SRP alone, and approximate 8% more sites obtained PD reduction ≥ 2 mm (*p* < 0.05). Meanwhile, the mean relative abundance of *Tannerella forsythia* was significantly lower in the combined treatment group (*p* < 0.05).

**Conclusion:**

The purified EGCG showed the potential to improve the outcome of periodontal non-surgical treatment and the new-type scaler tip provided an alternative vehicle for subgingival medication.

*Trial registration* The trial was registered in Chinese Clinical Trial Registry on 15 February 2020 (No.: ChiCTR2000029831, retrospectively registered). http://www.chictr.org.cn/showprojen.aspx?proj=49441.

## Background

Chronic periodontitis is an inflammatory disease initiated by plaque infection and imbalance of immune response in host defense system, resulting in breakdown of alveolar bone, attachment loss of junctional epithelium and eventually teeth loss. A substantial increase of gram-negative anaerobic rods, especially the red complex (*Porphyromonas gingivalis*, *Treponema denticola* and *Tannerella forsythia*) in the subgingival plaque acts a pathogenic role in the incidence and progression of this disease [1, 2]. Mechanical debridement as scaling and root planing (SRP) has been repeatedly demonstrated to be effective to remove subgingival pathogens and lead to an improvement of periodontal status [3, 4]. When dealing with deep periodontal pockets, surface irregularities and furcation area, complete removal of plaque and calculus is often proved difficult by SRP alone [5, 6], thereby providing a rationale for adjunct use of pharmacological therapy [7, 8]. A series of local anti-infective methods including oral rinses, subgingival irrigation and sustained-release or controlled-release vehicles is aimed at inhibiting periodontal pathogens and alleviate inflammatory response, and has been proved to enhance the outcome of non-surgical treatment [8–10].

Green tea catechins extracted from *Camellia sinesis* have been reported to possess an antibacterial effect against gram-negative anaerobic rods [11]. The growth and adherence of *P. gingivalis* onto the buccal epithelial cells could be completely inhibited by green tea catechins, especially EGCG exhibiting the strongest antibacterial activity [12]. Furthermore, EGCG could destroy established *P. gingivalis* biofilms and inhibited biofilm formation with the minimal inhibitory concentration of 0.5 mg/mL [13]. As anti-inflammatory reagents, tea extracts dose-dependently reduced the production of interleukin-6 and interleukin-8 in pathogen-stimulated epithelial cells [14], and EGCG suppressed the expression of metalloproteinases-9 in osteoblasts and the formation of osteoclasts in periodontal destruction [15]. Green tea catechins have been formulated into several forms of sustained-release medication to incorporate mechanical debridement, and demonstrated to be effective in improving its efficacy on periodontitis [16–18].

Local delivery of chlorhexidine as coolants of an ultrasonic scaler has been proposed, which was suggested to permit better access for its administration and might contribute to drug adsorption to root surface, however, the clinical benefits were still controversial [19–21]. Among studies above, conventional scaler tips were applied for medication, while drugs might fail to be delivered into the bottom of deep pockets via the supragingival outlets, thereby influencing drug effects on treatment outcome. In the present study, a new-type ultrasonic scaler tip was applied for subgingival scaling, through which drug solution could be ejected from the terminal outlet aligned to the bottom of periodontal pockets. Compared with conventional design, the new-type scaler tip could guarantee drugs to be delivered to the bottom, which might provide an alternative vehicle for subgingival medication. Green tea catechins which are readily resoluble in water could be prepared as cooling aqueous supply and medicated as above, however, the pertinent study was scarce up to now.

Hence, this study was designed to evaluate the clinical and microbiological efficacy of SRP using EGCG aqueous solution as coolants through the new-type ultrasonic scaler tip on chronic periodontitis.

## Methods

This split-mouth, randomized controlled clinical trial (Chinese Clinical Trial Registry, ChiCTR2000029831) of 6-month duration was conducted in the Department of Periodontology, Hospital and School of Stomatology, Peking University, China. All procedure followed the Consolidated Standards of Reporting Trials (CONSORT) guideline.

### Subjects selection

This study protocol was documentarily approved by the Ethics Committee of Peking University School of Stomatology (No. PKUSSIRB-201944047) and was conducted in accordance with the Helsinki Declaration of 1975, as revised in 2013. 20 patients were recruited into the study, depending on the following inclusion criteria: (1) patients diagnosed as generalized chronic periodontitis with CAL loss in > 30% of sites [22] and (2) patients systemically healthy, without any disease compromising wound healing. Exclusion criteria were as follows: (1) smoking, (2) pregnancy or lactation, (3) systemic antibiotics taken within previous 6 months, (4) subgingival scaling received within previous 6 months and (5) less than 10 maxillary teeth except for wisdom teeth. All periodontal sites associated with a PD ≥ 4 mm and bleeding on probing were selected as study sites in maxillary dentition of each subject, except wisdom teeth and teeth considered hopeless or to be extracted. Patients received a description of the study design and provided written informed consent before enrolled in the study.

### Ultrasonic equipment

**Fig. 1:**
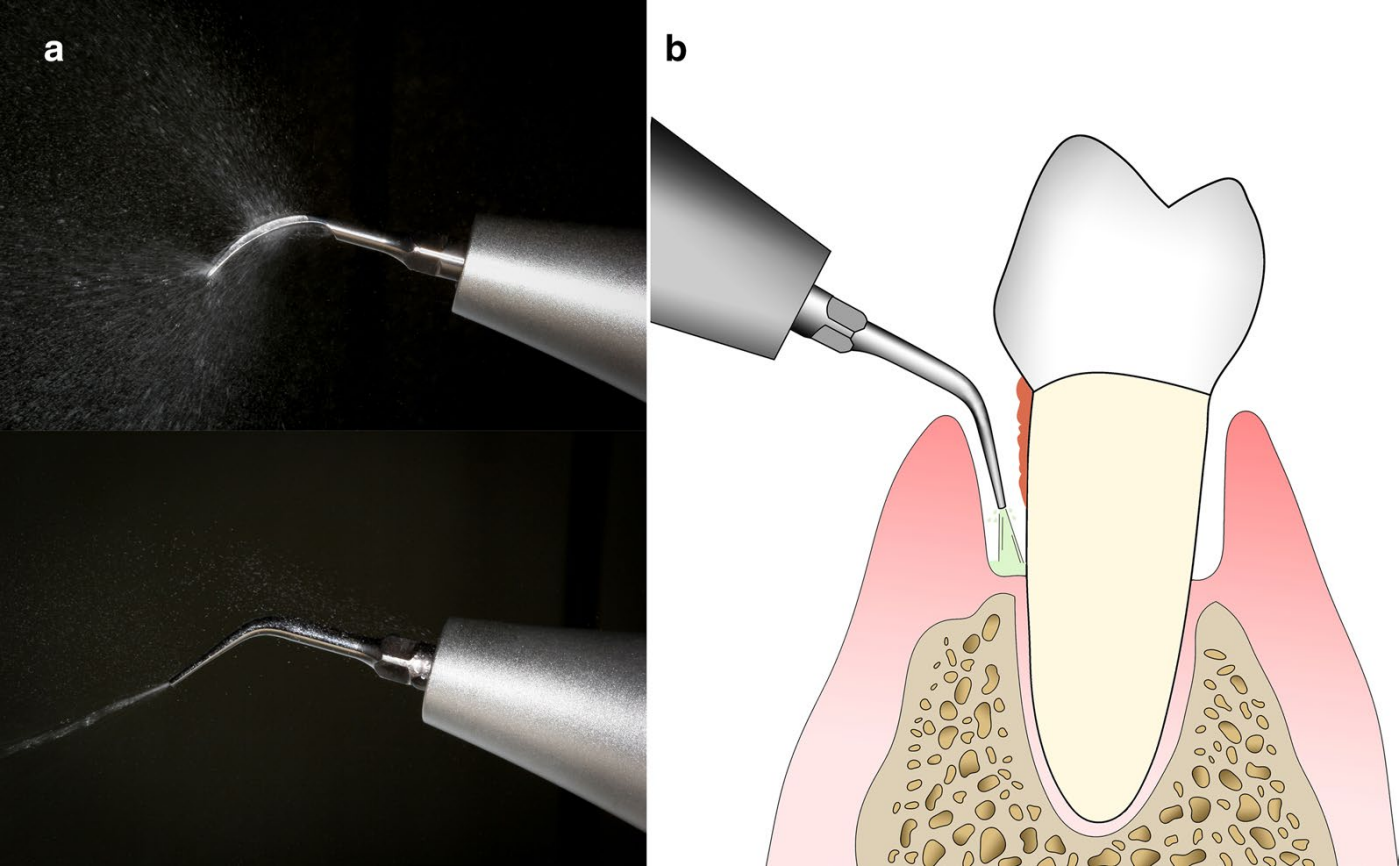
The ultrasonic scaler tips used in this study. **a** The conventional P3 (up) and new-type scaler tip (down), **b** terminal outlet of the new-type scaler tip allows coolants to be ejected into deep pockets

A piezoelectric ultrasonic scaler (SKL A7) fabricated two coolant containers was sponsored by SKL Medical Instrument Co. Ltd. (Guangdong, China), which could generate ultrasonic vibration at a frequency of 24–33 kHz. Two different subgingival scaler tips used in treatment represented a conventional design (P3, SKL, Guangdong, China) and a new-type terminal-outlet design (Patent No. ZL201820316091.3) which allows coolants to be delivered through the tip and ejected from the apex outlet in addition to ultrasonic vibration (Fig. 1). The flow rate of coolants (either water or EGCG solution) was turned up to maximum during ultrasonic scaling.

### EGCG testing and solution preparation

The EGCG used in this study was well-purified Sunphenon® EGCG purchased from Taiyo Green Power Co. Ltd. (Wuxi, Jiangsu, China). Determined by high performance liquid chromatography, the purity of this EGCG product was higher than 92%. Within 30 min before each treatment, 5 mg/mL EGCG aqueous solution was prepared with distilled water and stored in one labelled 500 mL coolants container, which was masked and with the cap tightened to avoid light and oxygen interference. The other labelled coolants container was filled with distilled water as control.

### Clinical examination

Primary outcome: PD and CAL were measured at 6 sites per tooth (disto-buccal, mid-buccal, mesio-buccal, mesio-lingual, mid-lingual and disto-lingual), using a Williams periodontal probe (Hu-Friedy®, Chicago, United States) with 1 mm increment. The discrepancy from the gingival margin and cemento-enamel junction to the base of the periodontal pockets was recorded to the nearest millimeter as PD and CAL, respectively.

Secondary outcome: bleeding index (BI, Mazza, 1981) and plaque index (PLI, Silness & Löe, 1964) were measured at 2 sites per tooth (buccal and lingual) and the scores from 0 to 5 and 0 to 3 were assessed respectively.

The intra-examiner reproducibility had been calibrated within 2 patients, 336 sites, prior to commencement of the measurement. The intraclass correlation coefficient (ICC) of PD and CAL was calculated as 0.969 and 0.959 respectively in duplicate measurements (n = 336), 30 min apart.

### Clinical protocol

Before enrollment, the randomized allocation of maxillary contra-lateral quadrants was determined with a coin-toss method in each subject by the investigator (Q.Y.). All patients received proper oral hygiene instruction and full-mouth supragingival scaling at the first visit. After 2 weeks, baseline recording of clinical parameters and microbiological sampling were performed by the examiner (Y.W.) who was blinded to the allocation. Then, the subgingival treatments were performed by the operator (J.Z.) and accomplished in 4 sessions arranged 1 week apart in each patient. As the split-mouth study design, the left or right maxillary quadrant sites with PD ≥ 4 mm was scaled and root planed with either EGCG solution or distilled water as coolants of the ultrasonic scaler. The sequence of each quadrant treatment was as follows: removing massive calculus and subgingival plaque with ultrasonic scaling (P3 Scaler Tip, SKL), thorough scaling and root planing to the bottom of periodontal pockets with hand instrumentation (Gracey Standard Curettes, Hu-Friedy®, Chicago, United States) until tooth surfaces were smooth and hard, then ultrasonic scaling at least 1 min per tooth (New-type Scaler Tip). Average 30–40 min was demanded for each quadrant where local infiltration anesthesia was conducted if necessary. The volume of either EGCG solution or distilled water consumed in each treatment was approximately 300–500 mL. The patients were informed to report if any discomfort related to the treatment was felt within 1 week.

The patients were recalled 3 and 6 months after treatment. All clinical parameters were recorded as baseline and microbiological samples were taken by the examiner (Y.W.), and the oral hygiene was reinforced when necessary. If there still existed study sites associated with PD ≥ 4 mm, the patients would receive ultrasonic scaling using the new-type scaler tip at a frequency of 24–33 kHz in accordance with the former allocation.

### Microbiological procedures

Based on the baseline clinical examination, a pair of study sites with PD ≥ 4 mm within contra-lateral premolars or the first molars were selected for subgingival plaque sampling. These selected sites were consistent among baseline, 3-month and 6-month re-evaluation. The subgingival plaque samples were collected by paper points method and stored at − 80 °C for microbiological testing. The PCR products of bacterial 16S rRNA were generated and sequenced after DNA isolation, as described in Liu et al. [23]. After annotated against the Human Oral Microbiome Database [24], the mean relative abundances of red complex pathogens were calculated in subgingival microbiome.

### Safety of treatments

The patients were instructed to report if any discomfort was felt during each treatment and 1 week after all treatment procedures. The adverse effects associated were as follows: (1) postoperative pain and dentinal hypersensitivity; (2) staining of tooth surfaces associated with medication; (3) subgingival emphysema related to the new-type scaler tip, which might manifest as noticeable swelling and crepitus of soft tissues; (4) fracture of the new-type scaler tip, which was monitored by checking post-treatment length and shape changes with the customized alginate impression of each labeled tip.

### Sample size calculation

A sample size of 20 patients was estimated according to the previous study which applied a sustained-release catechins mixture as an adjunct to SRP [18], thus 393 and 369 sites were obtained from 18 patients (2 drop-outs) in two groups respectively. Then a post hoc analysis at a two-side type I error of 0.05 (α) and 80% power of detection (1 − β) was conducted based on the primary outcome PD at month 6, determining that 287 sites per group would be necessary.

### Statistical analysis

The effect of different treatment groups on these clinical parameters at each interval was compared by Student’s *t* test. Within each group, the Bonferroni method (Paired-sample *t* test) was performed to compare the clinical parameters at different intervals. Pearson chi-square test was performed to compare the percentage of treatment sites between groups. Comparison of relative abundance in subgingival microbiome was performed with Wilcoxon signed rank test. Data processing and analysis was performed on SPSS 23.0 (IBM, Chicago, United States) and R (Stats package; R Foundation for Statistical Computing, Vienna, Austria). *p*-values < 0.05 were accepted for statistical significance.

## Results

**Fig. 2:**
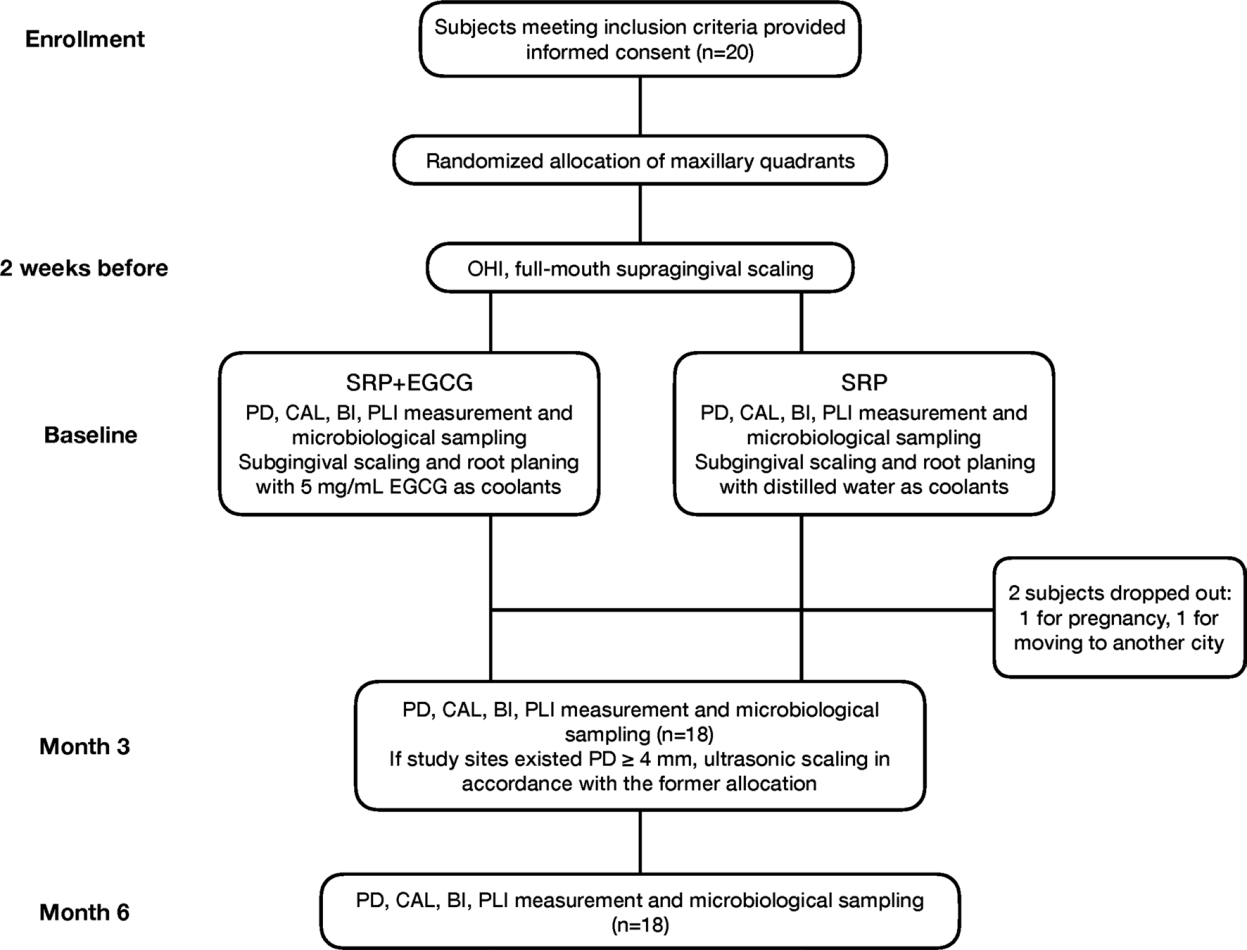
Flow diagram of the study

A total of 20 subjects (8 males, 12 females) who met the inclusion criteria were recruited in this study, however, 2 females dropped out at the 3-month follow-up due to pregnancy and moving to another city, respectively (Fig. 2). Thus, 18 patients (8 males, 10 females) aged 28–57 years completed the study, resulting in a total of 762 sites with baseline PD ≥ 4 mm brought into final  analysis. Table 1 presented the demographic characteristics of these subjects and baseline clinical parameters for all treatment groups (SRP with or without EGCG medication), and no statistically significant difference was determined between groups for any of the parameters.

**Table 1:** Demographic characteristics and clinical parameters (mean ± SD) at baseline

	Treatment groups	
	SRP + EGCG	SRP
Gender	8 males, 10 females	
Age (years)	37.6 ± 8.9	
Total number of sites	393	369
Number of sites in single-rooted teeth	249	223
Number of sites in multi-rooted teeth	144	146
PD (mm)	5.33 ± 1.38	5.28 ± 1.33
CAL (mm)	3.83 ± 2.18	3.75 ± 2.11
BI	2.83 ± 0.87	2.73 ± 0.81
PLI	0.78 ± 0.74	0.87 ± 0.70

No significant difference between groups for clinical parameters at baseline

### Clinical outcomes

**Fig. 3:**
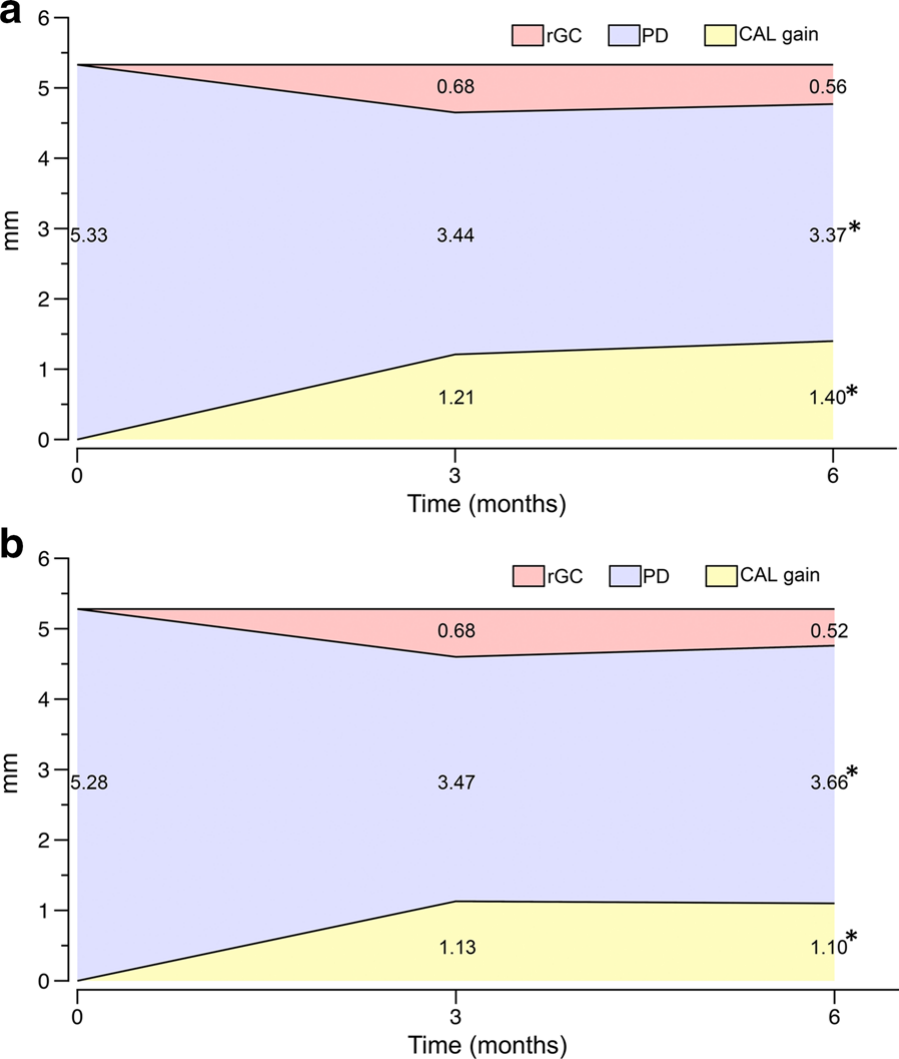
Mean PD, CAL gain and relative gingival recession (rGC) from baseline gingival margin changing over time. **a** sites treated with SRP plus EGCG and **b** sites treated with SRP alone (*p < 0.05, between-groups comparison with Student’s t test)

Table 2 showed the mean PD and CAL of sites treated with two clinical strategies at different intervals. At baseline, PD and CAL between the selected sites of two treatment groups were similar, 5.33 ± 1.38 mm versus 5.28 ± 1.33 mm and 3.83 ± 2.18 mm versus 3.75 ± 2.11 mm, respectively. After 3 months, both treatment groups showed statistically significant PD reduction and CAL gain, and SRP plus EGCG didn’t yield additional improvement in these parameters when compared with SRP alone (PD: 3.44 ± 1.29 mm versus 3.47 ± 1.3 mm, *p* = 0.696; CAL: 2.62 ± 2.16 mm versus 2.62 ± 2.18 mm*, p* = 0.985). However, the changes in PD over the next 3 months were discrepant between two treatment groups. Compared with month 3, PD in the SRP plus EGCG group continued to reduce till 6 months (3.37 ± 1.36 mm versus 3.44 ± 1.29 mm, *p* = 0.019), while the SRP alone group failed to obtain a further reduction, instead it presented a significant increase in PD (3.66 ± 1.43 mm vs 3.47 ± 1.3 mm, *p* = 0.001). Thus, at month 6, the study sites treated with SRP plus EGCG possessed a smaller PD parameter than those treated with SRP alone (*p* = 0.004). Regarding CAL changes during month 3–6, the scenario was similar that SRP plus EGCG contributed to a continuous gain of the clinical parameter (2.42 ± 2.11 mm vs 2.62 ± 2.16 mm, *p* = 0.005), which almost remained same in the SRP alone group (2.65 ± 2.09 mm vs 2.62 ± 2.18 mm, *p* = 0.674). The additional CAL gain of 0.30 mm from baseline was significantly in favor of the SRP plus EGCG group at month 6 (1.40 ± 1.55 mm vs 1.10 ± 1.73 mm, *p* = 0.011, Fig. 3a, b).

**Table 2:** Mean PD and CAL for sites treated with SRP or SRP plus EGCG

Clinical parameter		Mean ± SD			Within-group comparison
		Baseline	Month 3	Month 6	
PD (mm)	SRP + EGCG	5.33 ± 1.38	3.44 ± 1.29	3.37 ± 1.36	0 versus 3: *p* < 0.001* 0 versus 6: *p* < 0.001* 3 versus 6: *p* = 0.019*
	SRP	5.28 ± 1.33	3.47 ± 1.3	3.66 ± 1.43	0 versus 3: *p* < 0.001* 0 versus 6: *p* < 0.001* 3 versus 6: *p* = 0.001*
Between-group comparison		*p* = 0.656	*p* = 0.696	*p* = 0.004*	
CAL (mm)	SRP + EGCG	3.83 ± 2.18	2.62 ± 2.16	2.42 ± 2.11	0 versus 3: *p* < 0.001* 0 versus 6: *p* < 0.001* 3 versus 6: *p* = 0.005*
	SRP	3.75 ± 2.11	2.62 ± 2.18	2.65 ± 2.09	0 versus 3: *p* < 0.001* 0 versus 6: *p* < 0.001* 3 versus 6: *p* = 0.674
Between-group comparison		*p* = 0.611	*p* = 0.985	*p* = 0.139	

The comparison between groups at each interval was performed with Student’s *t* test (*p < 0.05)

The comparison within each group was performed with the Bonferroni method (*p < 0.05); 0, baseline; 3, month 3; 6, month 6

**Fig. 4:**
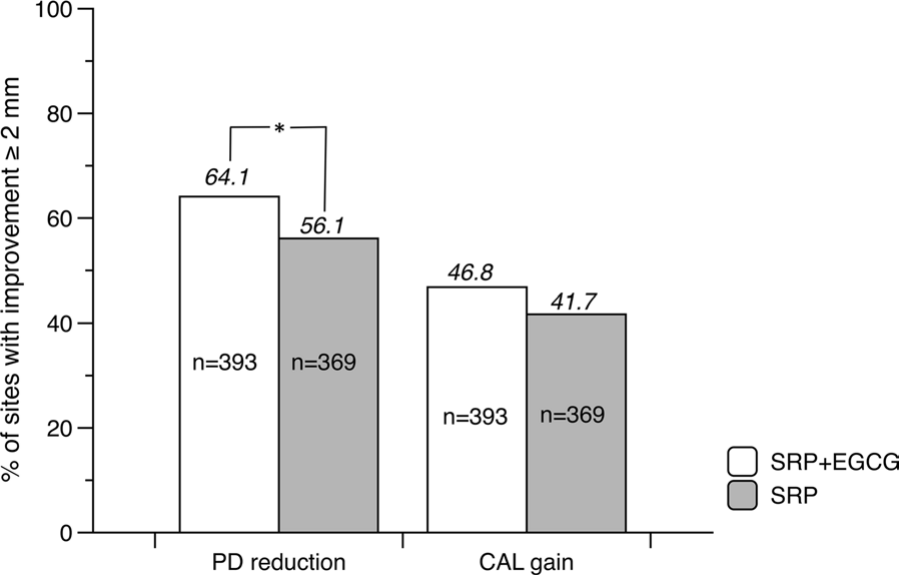
Percent in sites with PD reduction or CAL gain ≥ 2 mm at month 6. Comparison between SRP plus EGCG and SRP alone (*p < 0.05, Pearson chi-square test)

PD reduction after treatments could be ascribed to the corresponding gingival recession and gain of CAL. In this study, SRP plus EGCG medication obtained a greater PD reduction than SRP alone at month 6, while relative gingival recession (rGC) from the baseline level was similar within two treatment strategies, which indicated that an additional gain of CAL mainly potentiated the PD reduction (Fig. 3a, b). After 6 months, the difference of PD reduction was 0.33 mm between groups (1.96 ± 1.38 mm vs 1.63 ± 1.38 mm, *p* = 0.001) and the additional CAL gain was 0.30 mm favored the SRP plus EGCG group. Considering a clinically meaningful improvement, the site percentages of PD reduction or CAL gain ≥ 2 mm at month 6 within all selected sites were calculated, as presented in Fig. 4. A significant 8% favored the SRP plus EGCG group was observed in the PD reduction ≥ 2 mm at month 6 (64.1% vs 56.1%, *p* < 0.05). Meanwhile, more sites of approximately 5.1% were found to obtain CAL gain ≥ 2 mm at month 6 in the SRP plus EGCG group, though this difference was not statistically significant (46.8% vs 41.7%).

Compared with baseline examination, both treatment strategies lead to the reduction of BI and PLI at month 3 and 6, and no further improvement occurred during 3–6 month. Results of between-group comparison at each interval didn’t show a significant discrepancy (data shown in the Additional file 1).

### Microbiological outcomes

**Fig. 5:**
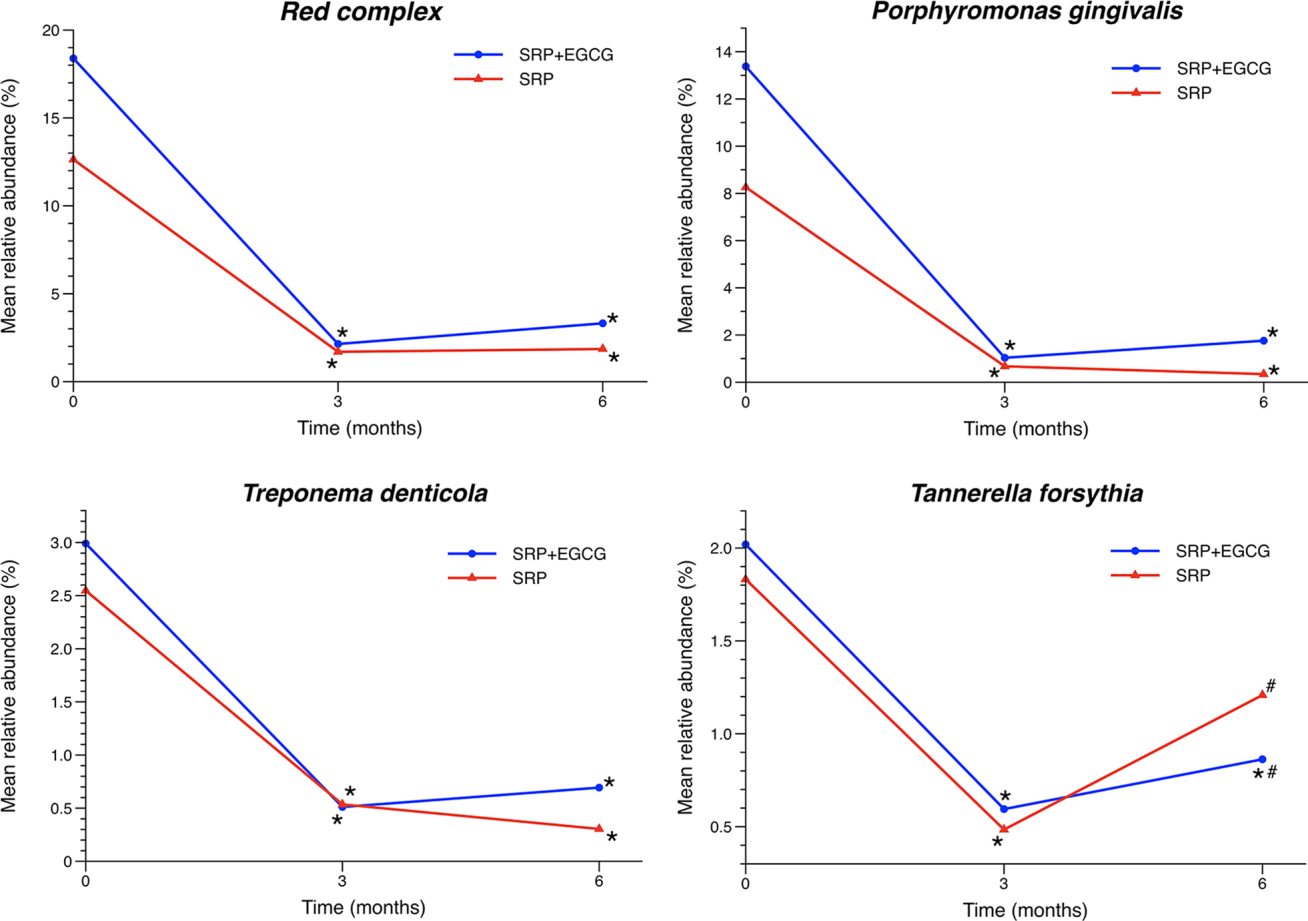
Mean relative abundance of red complex pathogens. Within-group and between-group comparison using Wilcoxon signed rank test (*p < 0.05, month 3, 6 vs baseline; ^#^*p* < 0.05, SRP plus EGCG vs SRP)

The mean relative abundances of red complex pathogens and individual pathogens were presented in Fig. 5. Before treatment, the red complex pathogens occupied the major part of subgingival microbiome, mean 18.4% and 12.6% in two groups respectively, while they were declined to < 5% by either of the treatment strategies with no significant difference between groups. As for individual pathogens, the species of *P. gingivalis* and *T. denticola* decreased and maintained at the relatively low level consistently, however, no significant difference was observed between groups. The mean relative abundance of *T. forsythia* was also reduced, while it tended to revert to the baseline level in SRP group, thus resulting in a significant difference between groups at month 6.

### Safety

Most patients fed back tooth sensitivity and gingival pain within tolerance instantly after treatments, which could be relieved gradually in 1–2 weeks and were similar between treatment groups. The staining of tooth surfaces was evaluated at each visit and no significant discrepancy was observed between groups. During the whole procedure, no patient appeared noticeable swelling of soft tissues associated with subgingival emphysema. A total of two new-type scaler tips were applied in this study, which appeared no length and shape changes after 43 and 55 times, approximate 1290 and 1650 min respectively.

## Discussion

As coolants, chlorhexidine has been delivered through conventional scaler tips during SRP, however, the clinical benefits were still controversial [19–21]. Nosal et al. [25] evaluated the penetration range of the dye-colored coolant into periodontal pockets of teeth to be extracted and found that through conventional scaler tips the dyed root surface was along full extent of the penetration path, however, had very little lateral dispersion. The teeth to be extracted always have quite loosen periodontal pockets with severe inflammation, while the pockets were relatively tight in teeth to be treated and the penetration range and depth might be limited, thus influencing drug effects. In this study, we applied a new-type scaler tip with the terminal outlet, which could guarantee enough range of subgingival medication and local drugs to be delivered to the bottom of periodontal pockets. Meanwhile, the transport of therapeutic agents towards local tissues might be enhanced in conjunction with ultrasonic cavitation and acoustic microstreaming [26]. Considering the possible subgingival emphysema caused by the new-type scaler tip, the conventional scaler tip was still used first to remove the massive calculus and release the tight periodontal pockets.

As anti-microbial and anti-inflammatory, green tea catechins have been formulated into sustained-release drug, such as gel and strips, as an adjunct to mechanical debridement, while most of them focused on the short-term efficacy less than 3 months [11, 16, 17, 27]. Meanwhile, the extracted catechins mixture was applied to prepare these sustained-release drugs. According to microbiological studies, EGCG exhibited better anti-microbial and anti-inflammatory than other main catechins in green tea extract, therefore, we applied a 5 mg/mL EGCG solution as coolants, which was higher than its minimal inhibitory concentration to periodontal pathogens [13]. The pertinent study applying purified EGCG medication on periodontal disease was absent, while our previous study has proved that the concentration as high as 5 mg/mL could maintain stable under ultrasonic vibration [28].

The split-mouth controlled design was adopted to balance the clinical disease characteristics, such as extent of periodontal inflammation and sufficient site number selected, and the patient-level factors influencing the treatment efficacy between two groups. Only sites with PD ≥ 4 mm in the maxillary dentition were recruited into this study to avoid the possible drug interference via saliva of oral floor, and the dental suction was applied during ultrasonic scaling. After either treatment strategy, the significant PD reduction and CAL gain were obtained at month 3, as 1.8 mm and 1.2 mm respectively, which were in accord with the reported research associated with efficacy of non-surgical treatment on chronic periodontitis [29]. However, no significant difference in clinical parameters was found between the treatments of SRP plus EGCG and SRP alone at 3-month follow-up. Thus, the selected sites remained PD ≥ 4 mm received repeated ultrasonic scaling using the new-type scaler tip with or without EGCG medication as the allocation before. During the next 3 months, the PD and CAL continued to be improved in the SRP plus EGCG group, while the scenario was different in sites treated SRP alone that these two clinical parameters tended to rebound, thus presenting a significant difference of PD reduction as 0.33 mm and CAL gain of 0.30 mm between groups. Meanwhile, the clinical significance of two treatment strategies was tested with the 2 mm threshold, which was regarded as a clinical level to monitor the disease progression and evaluate the treatment success, and also avoid examination bias of the periodontal probe with 1 mm increment [9]. Compared with SRP alone, a significantly 8% more sites could obtain the PD reduction ≥ 2 mm when treated with additional EGCG medication. Within sites treated with SRP alone in this study, the longitude tendency of PD and CAL was to rebound, which was also observed in previous studies related to mechanical treatment on chronic periodontitis [30], while the efficacy was maintained with an additional EGCG medication. Among previous studies, only Rattanasuwan et al. [18] reported the study focused on the 6-month longitude efficacy of sustained-release catechins mixture gel on periodontitis, in which PD and CAL also presented a tendency to rebound after 3-month, but no significant discrepancy was found between treatments with or without catechins medication. Although both sustained-release medication and the new-type scaler tip could deliver drugs to the bottom of deep pockets, even the longer acting duration within the former, the combined effect of EGCG and ultrasonic cavitation might be one of the reasons on clinical difference between the study above and ours.

The red complex pathogens were recognized as the most important pathogens associated with periodontitis, which significantly decreased to mean relative abundance < 5% in subgingival microbiome after either treatment, proving the microbial analysis method applied was reliable in this study. However, the analysis of the red complex and *P. gingivalis* didn’t show significant differences between the treatments of SRP plus EGCG and SRP alone, and only the pathogen *T. forsythia* showed a significant difference favored the combined EGCG medication. Hattarki et al. [17] reported the sustained-released catechins mixture strips presented an additional antimicrobial effect on *T. forsythia* at 5-week and *P. gingivalis* at 1-week. The purified EGCG exhibited a potent anti-microbial effect on *P. gingivalis* biofilms in vitro, while no additional significant difference was found in our in vivo study, which might be ascribed to the relatively shorter exposure time of EGCG solution in the subgingival environment [13]. However, through repeated medication at 3-month follow-up, an additional microbial effect on *T. forsythia* was found at month-6, which was also observed in the previous study [17]. The antimicrobial effect of purified EGCG in vivo needs more clinicomicrobiological studies to verify  in the future. In the present study, the additional PD reduction mainly ascribed to the gain of CAL, which might indicate that the anti-inflammatory effects of EGCG on periodontal tissues and the new-type scaler ejecting the drug towards the bottom of deep pockets could contribute the formation of epithelial attachment [14, 15].

This study focuses on the long-term efficacy of purified EGCG delivered by the new-type scaler tip, while its effect within 3 months also deserves to be investigated. Furthermore, as another liquid drug, chlorhexidine could exert antibacterial effect and has been applied in periodontal treatments for decades, which also has the potential to be delivered by the new-type scaler tip. Based on our primary results and previous studies, multicenter trial with large sample size should be performed to verify the efficacy of purified EGCG or green tea catechins as periodontal medication and compare with other drugs such as chlorhexidine in the future. For the rational comparison within similar subjects, this study recruited patients with generalized chronic periodontitis which was consistent with previous studies [16–18]. As the new international classification of periodontal diseases has been proposed in 2018 [31], 18 patients into the final analysis could be diagnosed as generalized periodontitis (stage II or III, grade B) based on their baseline examination, which could provide a reference for future studies using this new classification system.

## Conclusions

The purified EGCG showed the potential to improve outcome of non-surgical periodontal treatment and the new-type scaler tip provided an alternative vehicle for subgingival medication.

## Supplementary Information

**Additional file 1**. Mean BI and PLI for sites treated with SRP or SRP plus EGCG.

## Data Availability

The datasets used and/or analyzed during the current study are available from the corresponding author on reasonable request.

## Abbreviations

EGCG: (−)-Epigallocatechin gallate
SRP: Scaling and root planing
PD: Probing depth
CAL: Clinical attachment level
BI: Bleeding index
PLI: Plaque index
ICC: Intraclass correlation coefficient
SD: Standard deviation
rGC: Relative gingival recession
