# Supplementary material for: Efficacy of (−)-epigallocatechin gallate delivered by a new-type scaler tip during scaling and root planing on chronic periodontitis: a split-mouth, randomized clinical trial
Source: BMC Oral Health. 2021 Feb 18;21:79. doi: 10.1186/s12903-021-01418-1 (PMC7890979; doi:10.1186/s12903-021-01418-1)
Supplement: Supplementary file 1 — Additional file 1. Mean BI and PLI for sites treated with SRP or SRP plus EGCG. [file 12903_2021_1418_MOESM1_ESM.docx]

**Additional file 1**

**Table: Mean BI and PLI for sites treated with SRP or SRP plus EGCG**

| Clinical parameter | | Mean ± SD | | | Within-group comparison |
| --- | --- | --- | --- | --- | --- |
|  |  | Baseline | Month 3 | Month 6 |  |
| BI | SRP + EGCG | 2.83 ± 0.87 | 1.84 ± 0.70 | 1.79 ± 0.78 | 0 *vs.* 3: *p* < 0.001* 0 *vs.* 6: *p* < 0.001* 3 *vs*. 6: *p* = 0.378 |
|  | SRP | 2.73 ± 0.81 | 1.91 ± 0.78 | 1.86 ± 0.75 | 0 *vs.* 3: *p* < 0.001* 0 *vs.* 6: *p* < 0.001* 3 *vs.* 6: *p* = 0.425 |
| Between-group comparison | | *p* = 0.253 | *p* = 0.376 | *p* = 0.423 |  |
| PLI | SRP + EGCG | 0.78 ± 0.74 | 0.52 ± 0.61 | 0.48 ± 0.58 | 0 *vs.* 3: *p* < 0.001* 0 *vs.* 6: *p* < 0.001* 3 *vs.* 6: *p* = 0.395 |
|  | SRP | 0.87 ± 0.70 | 0.53 ± 0.62 | 0.60 ± 0.65 | 0 *vs.* 3: *p* < 0.001* 0 *vs.* 6: *p* < 0.001* 3 *vs.* 6: *p* = 0.169 |
| Between-group comparison^α^ | | *p* = 0.611 | *p* = 0.985 | *p* = 0.139 |  |

The comparison between groups at each interval was performed with Student’s *t* test (**p* < 0.05).

The comparison within each group was performed with the Bonferroni method (**p* < 0.05); 0, baseline; 3, month 3; 6, month 6.
